# Application of CRISPR/Cas9 technology in the modeling of Gaucher disorder

**DOI:** 10.1016/j.bbrep.2024.101872

**Published:** 2024-11-19

**Authors:** Mehran Reyhani-Ardabili, Mohadeseh Fathi, Soudeh Ghafouri-Fard

**Affiliations:** Department of Medical Genetics, Shahid Beheshti University of Medical Sciences, Tehran, Iran

**Keywords:** Gaucher disease (GD), GBA1, CRISPR

## Abstract

Gaucher disease (GD) is a metabolic disorder caused by mutations in the *GBA1*, located on 1q22. This gene encodes glucocerebrosidase (glucosylceramidase) enzyme. GD has a wide range of clinical manifestations from a perinatally lethal type to an asymptomatic form. While different types of targeted therapies and hematopoietic stem cell transplantation have been suggested as therapeutic options for patients with GD, success rates were not optimal. Recent advance in the CRISPR technology has raised the hope for treatment of metabolic disorders such as GD. This technology has also facilitated identification of the molecular mechanisms underlying pathologic events in this disorder. The current review addresses both mentioned aspects of application of CRISPR technology in the field of GD.

## Highlights

• Gaucher disease (GD) is a metabolic disorder caused by mutations in the *GBA1* gene.
• Different types of targeted therapies and hematopoietic stem cell transplantation have been suggested as therapeutic options for patients with GD.
• CRISPR technology has raised the hope for treatment of GD.
• This technology has also facilitated identification of the molecular mechanisms underlying pathologic events in GD.

## 1 Introduction

Gaucher disease (GD) includes a range of clinical manifestations from a perinatally lethal type [1] to an asymptomatic disorder [2]. This disorder is classified into three main clinical types (1–3) and two clinical subtypes (perinatal-lethal and cardiovascular). These types of classification have practical significance for determination of prognosis and treatment options [3]. Type 1 GD is manly associated with different manifestations in bone (osteopenia, focal lytic or sclerotic lesion, and osteonecrosis), in addition to hepatosplenomegaly, anemia, thrombocytopenia and lung disorder, but absence of primary central nervous system (CNS) involvement [4]. In type 2 GD, CNS involvement starts before two years old with restricted psychomotor development. The disease has a rapid progressive course leading to demise by age of two to four years [5]. Finally, in type 3 GD, CNS disease has a childhood onset with a more gradually progressive course, and longer survival [6].

GD is diagnosed based on the observed defects in the activity of glucocerebrosidase (glucosylceramidase) enzyme in the peripheral blood leukocytes, or through detection of biallelic pathogenic variants in *GBA1* gene, located on 1q22 [7]. Deficiency in the activity of this enzyme results in progressive accretion of glucosylceramide and glucosylsphingosine inside the lysosomes, particularly in the reticuloendothelial system [8]. Accumulation of glucosylceramide in macrophages prompts their conversion into engorged cells recognized as Gaucher cells. Infiltration of these cells into bone marrow, spleen and liver is regarded as the main mechanism of development of clinical features of GD, including hepatosplenomegaly, thrombocytopenia, anemia and coagulation disorders [9,10]. Another gene, namely *GBA2* has been found to encode an enzyme with glucocerebrosidase activity. Thus, screening for *GBA2* mutations should be considered in suspected patients [11].

Both enzyme replacement [12,13] and substrate reduction therapies with miglustat [14] or eliglustat [15,16] have been employed as targeted therapies for GD. In addition, hematopoietic stem cells transplantation has been suggested as a therapeutic option for patients with severe GD, particularly those with chronic CNS involvement (type 3 GD) [17]. Recent advance in the Clustered Regularly Interspaced Short Palindromic Repeats (CRISPR) technology has raised the hope for treatment of metabolic disorders such as GD. This technology has also facilitated identification of the molecular mechanisms underlying pathologic events in this disorder. The current review addresses both mentioned aspects of application of CRISPR/Cas9 technology in the field of GD.

## 2 CRISPR technology in the modeling of GD

**Fig. 1:**
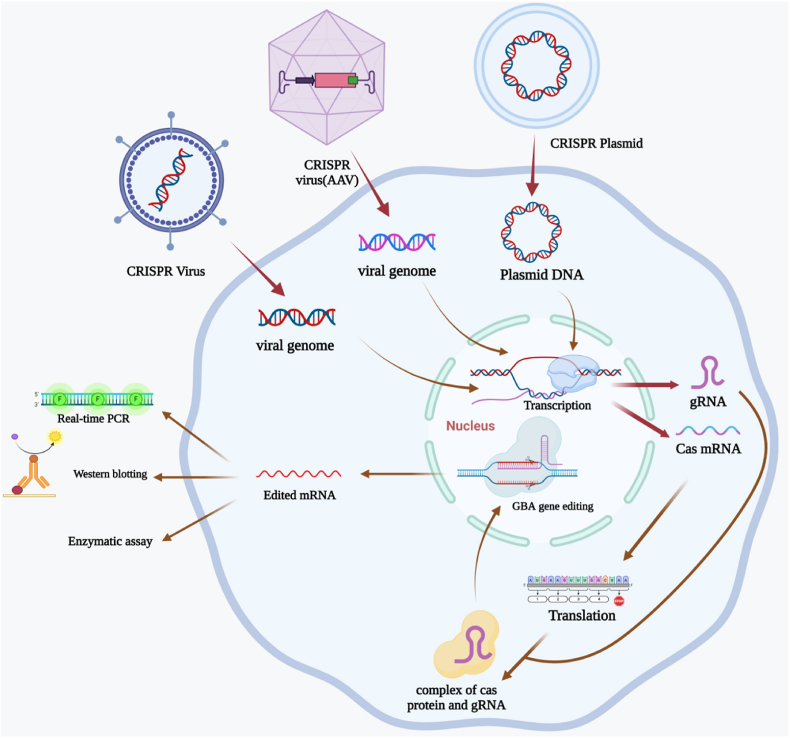
An overview of steps and modalities used for disease modeling using CRISPR technology. Cellular and animal models of Gaucher disease have been created using CRISPR/Cas9 technology. Different delivery methods, including viral and plasmid vectors have been used for this purpose. The results have been confirmed using real-time pCR, Western blotting and enzyme assay (AAV: adeno-associated virus; Cas: CRISPR-associated nuclease; GBA1: Glucosylceramidase Beta 1; gRNA: guide RNA).

At least six cellular models as well as four animal models of GD have been produced by different groups using CRISPR/Cas9 editing technology (Table 1). Fig. 1 shows an overview of steps and modalities used in for disease modeling using CRISPR technology.

**Table 1:** CRISPR technology in the modeling of Gaucher disorder (KO: knock out).

Cell line	Animal model	CRISPR-Cas9-Targeted Gene (s)	Targeted site of gene	Mutation type	CRISPR-Cas9 delivery/vectors	Verification of the GBA1 gene disruption	Other verification tests	Enzyme Activity	Cells morphology/features after editing with CRISPR	Treatment	References
HEK 293	–	*GBA1*	Exon 3	–	sgRNA, TrueCut™ Cas9 Protein v2, Lipofectamine™ Cas9 plus reagent, Lipofectamine™ CRISPRMAX™ reagent	DNA sequencing, Western blotting	Endo-H and Endo-F Digestion, Enzymatic Activity, Quantitative Real Time PCR, LysoGL1 Measurement Cytokine Measurement, Annexin-V Staining, Lactate Dehydrogenase (LDH) Activity	–	–	–	[18]
Glioblastoma U87 cells (U87)				deletion of the whole *GBA1* exon 2 and the first part of exon 3, compound heterozygosis with an in frame six base pair deletion in exon 3				<1 %	In frame six base pair deletion leads to a protein lacking in Thr82 and Arg83. The mutant glucocerebrosidase is unfolded and retained in ER. Adding LPS and ATP, showed a higher elevation of IL-1β synthesis, but no production of TNFα or IL-6. Significant increase of α-syn abundance led to cell death.	–	
THP-1				in-frame exclusion of exon 3 and exons 3/4				= 1 %	Expression of two proteins of ∼53 and 48 kDa	–	
THP-1	–	*GBA1*	Exon 3	Two large in-frame deletions in the catalytic domain (c.115 + 145_307+1del and c.246_441del).	–	Osteoclast Differentiation Assay, Enzymatic Activity,	Cytokine Measurement, Glucosylsphingosine Measurement (GlcSph)	<1 %	IL-1β provokes GBAKO-THP1 cells to differentiate osteoclasts higher than the THP-1 WT cells. GBAKO cells showed higher levels of IL-1β and TNF-α. There was no significant difference in the levels of IL-6 released by GBAKO-THP1 cells compared to WT cells.	rhGCase increases enzyme activity, but ABX has no significant effect on enzyme activity. Significant reduction in substrate accumulation in cells treated by ABX, rhGCase and SRT; All treatments (rhGCase, ABX, SRT and PPS) effectively decreased osteoclastogenesis in GBAKO-THP1 cells.	[19]
HEK293 cells	partially humanized GD mouse model	*GBA1*	Exons 5-7	F213I mutation	Lentivirus expressing CAS9 protein and sgRNA of GBA1, PCDH- immediate early enhancer and promoter (CMV)-HA plasmid	Western blotting, Histological Analysis	–	–	–	–	[20]
HAP1	–	*GBA*	Exon 6	An insertional (479 bp) mutation and premature termination of GBA (frameshift)	–	PCR and double-stranded DNA sequencing, GBA Enzyme Assay	Lysosome Staining (LysoTracker), Fluorescent and Confocal Microscopy	4.3 %	HAP1 cells, after two passages of revival and culturing, display a fibroblast-like appearance and adhere to the surface. GBA-KO line and the HAP1 parental control showed no significant disparities in cell morphology and growth behavior. GBA-KO cells had higher lysosome count compared to the parental control cells.	Presence of recombinant human GBA, increased GBA levels in GBA-KO cells in a dosage-dependent manner.	[21]
hFB	–	*GBA2*	Exons 1 and 6	84G ins, N370S, L444P, A456P, V460V, V394L, P415R, R257Q, R120W, F213I,	pcDNA3.1, pcDNA6, pET21a	Quantitative real-time PCR, Protein preparation	Western blotting analysis, Glucosidase activity assay	2.6 %, 5.6 %, 8 % for types I, II, and III of Gaucher fibroblasts, respectively	GBA2 activity is suppressed in the absence of GBA1 activity, indicating a dependency of GBA2 function on GBA1. The structural prerequisites for the inhibition of GBA2 by sphingosine have been elucidated. Sphingosine can reversibly impede GBA2 activity through a mixed-type inhibition mechanism. Sphingosine acts as an inhibitor of GBA2 in Gaucher cells, effectively halting the accumulation of sphingosine and thereby mitigating cytotoxic effects.	–	[22]
HAP1				479-bp insertion in exon 6, 1-bp insertion in exon 6, 1-bp insertion in exon 1, 5-bp deletion in exon 1							
BE(2)-M17 cell line	–	*GBA*	Exon 3	N370S and L444P[23]	pSpCas9n(BB)-2A-GFP (PX461) plasmid, pCMV3-hGBA-N370S and pCMV3-hGBA-L444P plasmids	Sphingolipid quantification, Total homogenate and subcellular fractionation from cells, Immunoblot detection	Filter retardation assay, Extracellular synuclein detection, Cellular proteolytic assays, Lysosomal enzymatic activities, Lysosomal pH measurement, qPCR, Endoglycosidase-H sensibility assay, Cellular viability, Immunofluorescence assessment of LAMP-2A mRNA, Cholesterol quantification	7.66 % and 15.85 % for GBA N370S and GBA L444P lines, respectively	The deficiency in glucocerebrosidase function results in significant disruption within lysosomes, causing a cascade effect that impairs the activity of other lysosomal enzymes, destabilizes lysosomal membranes, alters intralysosomal pH levels, and facilitates the buildup of sphingolipids and cholesterol within lysosomes. These localized processes ultimately hinder autophagy mechanisms, notably chaperone-mediated autophagy, which is the primary pathway for degrading α-synuclein.	–	[23]
–	Drosophila	*Gba1*	Intron 3	The CRIMIC cassette, containing SA-T2A-GAL4-PolyA (from Gal4), was inserted into the intron 3 of Gba1b.	VECTASHIELD	Immunostaining	ERG recording, Transmission electron microscopy, Coculture assay, Conditional ACSF media incubation assay	30–40 %	Gba1b expression is localized in glial cells rather than neurons, with its loss resulting in abnormal glial morphology and progressive neurodegeneration. The absence of Gba1b leads to light/activity-dependent accumulation of GlcCer in neurons and glial cells. Loss of function of the white gene exacerbates GlcCer accumulation and neurodegeneration. Mammalian cell coculture assays showed the transport of GlcCer from neurons to glial cells.	–	[24]
–	Zebrafish	*gba2*	Exon 1	Germ-line transmitted deletion of 31 bp in the splice-site region of exon 1.	pDEST-ubi:hGBA	Activity-based probes, quantification of glycolipid metabolites	(Glyco)sphingolipid analysis, Western blot and total protein staining, LC-MS/MS, fluorescence scanning, Labeling of β-glucosidases with ABPs, Injection of gba1−/− fish with Cerezyme	40 %	Deactivating Gba1 increased GlcSph levels, while inhibiting GlcCer synthase in Gba1-deficient larvae decreased GlcCer and GlcSph. Simultaneous inhibition of GCS and Gba2 decreased excessive GlcChol levels, and overexpressing human GBA1 or injecting recombinant GBA1 lowered GlcSph levels.	–	[25]
–	Zebrafish	mir-155, *gba1*	Hyp188III site	55bp deletion, large insertion (253bp) with small (5bp) deletion	–	qPCR, TaqManTM MicroRNA Assay	IHC	<9 %	Up to 12 weeks post-fertilization, gba1−/− zebrafish appear visually similar to their wild-type siblings. Deleting miR-155 did not affect the lifespan or the development of abnormal motor function in gba−/− zebrafish. In gba1−/−zebrafish, there was significant microglial accumulation in the optic tectum and cerebellum, which was not improved by miR-155 genetic ablation.	–	[27]
	medaka (Oryzias latipes)	*GBA2*	Exon 5	21 bases deleted and 2 bases inserted.	pCS2+hSpCas9, pcDNA3-mock, pcDNA3p62.	The GBA2 enzymatic activity assay, Locomotor function analysis, Immunoblotting analysis, Immunohistochemical analyses	Cell counting, qRT-PCR, LC–ESI–MS/MS for the sphingolipid profle analysis	∼58 %	Deletion of Gba2 in medaka lacking gba1 led to an increase in glucosylceramide accumulation without any improvement in the pathological changes associated with Gaucher disease, asyn accumulation and swimming defects. While gba2 KO medaka did not exhibit visible phenotype, biochemical examination detected asyn accumulation in the brain. gba2 KO medaka displayed a tendency towards elevated sphingolipids in the brain.	–	[26]

Pavan et al. used CRISPR/Cas9 for establishment of cellular models imitating GD manifestations. In fact, they presented two GD cellular models through editing *GBA1*. Following development of an editing workflow using human embryonic kidney (HEK) cells, they edited monocytic cell line THP-1 and glioblastoma U87 cells for development of GD monocyte and glia, respectively [18]. Both mutant cell lines exhibited down-regulation of mutant acid β-glucosidase levels accompanied by immense accumulation of substrate. Besides, experiments in the U87 mutant cells revealed retention of the mutant enzyme in the ER and subsequent proteasomal degradation the enzyme, prompting unfolded protein response. These cells also exhibited elevated levels of IL-1β, independent of inflammosome activation, α-synuclein accretion and high rate of cell death in compared with wild-type cells. Cumulatively, the cellular models of GD generated by this group presented a practical model for understanding the pathophysiology of GD and conduction of high-throughput drug screening studies [18]. Another isogenic model of GD monocytes was developed by Ormazabal et al. [19]. These cells exhibited elevation of production of proinflammatory cytokines, namely IL-1β and TTNF-α and induction of osteoclastogenesis. Notably, increased osteoclastogenic potential of GD monocytes did not depend on their interactions with the GD niche or other GD cells. Most remarkably, author showed restoration of GD-related changes after treatment of these cells with a variety of therapeutic options, including recombinant human β-glucocerebrosidase, a substrate synthase inhibitor, a pharmacoperone, and an anti-inflammatory substance, approving the suitability of this model for conduction of high-throughput screening of therapeutic substances [19].

Guo et al. established the GD mice model of incompletely humanized *Gba1* gene with F213I mutation, a common mutation among Chinese patients. *In vitro* assessment of β-glucocerebrosidase activity revealed that the product of partially humanized *Gba1* gene, in which the mice exons 5–7 were replaced by the equivalent human exons, had comparable activity with the wild-type mice *Gba1*, whereas the F213I mutation in the humanized *Gba1* resulted in the reduction of enzyme activity. Authors also used ES cell targeting to create animals expressing the partially humanized *Gba1*-F213I. Homozygous *Gba1* ^F213I/F213I^ mice exhibited significant reduction in the β-glucocerebrosidase activity and expired within a day after birth. Notably, their epidermal stratum corneum was abnormal [20].

Brown et al. used CRISPR/Cas9 technology to create an insertional mutation and premature termination of *GBA* in HAP1 cells. GBA knockout was confirmed by both genotyping and enzymatic assays. While *GBA*-knockout cells exhibited no significant change in cell morphology and growth, they had higher numbers of granular lysosomes in their cytosols compared to their parental controls. This model could be used to assess therapeutic effect of recombinant human enzyme [21].

Schonauer et al. used CRISPR/Cas9 technique to generate GBA2-deficient human fibroblast-like cells in order to assess the role of GBA2 in GD pathogenesis and its relation with GBA1. Using this technique, they reported a GBA1-dependent decrease in GBA2 activity in GD. Moreover, they proposed a negative feedback loop, in which sphingosine suppresses GBA2 activity in GD cells, inhibiting additional accretion of sphingosine and, thus limiting its cytotoxicity [22].

In order to find the relevance and mechanism of involvement of *GBA* in the pathogenesis of Parkinson's diseases, Navarro-Romero et al. established a number of differentiated and stable human dopaminergic cells that have the two most common *GBA* mutations. In addition, they generated *GBA* knockout cell lines as models for investigation of the impact of these mutations on accumulation of α-synuclein. They reported that loss of β-glucocerebrosidase activity results in lysosomal dysfunction, stimulating defects in the activities of other lysosomal enzymes, disturbing lysosomal membrane stability, and inducing intralysosomal accretion of sphingolipids and cholesterol. These pathologic events results in the defects in the autophagy pathway, principally chaperone-related autophagy, which has a role in the degradation of α-synuclein. Using this modeling system, authors showed the importance of lysosomal functions and lipid metabolism in the pathogenesis of Parkinson's disease [23].

Animal models expressing mutant *GBA* variants have also been generated. For instance, Wang et al. produced a null allele of the Drosophila ortholog *Gba1b* through insertion of the Gal4 using CRISPR/Cas9 method. They showed expression of Gba1b in glial cells but not in neurons. Glial-specific knockdown mimicked the abnormalities found in Gba1b mutants. Notably, these defects were rescued through enhancement of expression of human GBA1 in glial cells. Further experiments showed that glucosylceramide is produced upon neuron activity, being transferred from neurons to glial cells by exosomes. The latter process was enhanced by glial TGF-β/BMP [24].

Lelieveld et al. used this technique in zebrafish in order to investigate the effect of Gba2 in glycosphingolipid metabolic pathways during Gba1 deficiency. CRISPR/Cas9 was used to develop gba1−/−, gba2−/−, and gba1−/−:gba2−/− zebrafish knockouts. Notably, both up-regulation of human GBA1 and injection of recombinant GBA1 were shown to decrease glucosylsphingosine. Taken together, authors suggested zebrafish larvae as an appropriate model for investigation of glucosidase action in glycosphingolipid metabolism *in vivo* [25]. In another study, CIRSPR/Cas9 technique was used to examine the effects of Gba2 deletion on the phenotype of gba1 knockout medaka, showing exacerbation of glucosylceramide accretion and no amendment of neuronopathic GD pathological alterations, α-synuclein accumulation, or swimming defects. Therefore, deletion of Gba2 did not amend the pathological alterations or behavioral defects of gba1 knockout medaka, and GBA2 affects α-synuclein accumulation in the brain [26].

## 3 CRISPR/Cas9 technology in the management of GD

Two independent studies have used CRISPR/Cas9 technology for correction of *GBA* mutations (Table 2). An efficient CRISPR/Cas9-based method was used by G. Scharenberg et al. in human hematopoietic stem and progenitor cells. This method aimed to target glucocerebrosidase expression cassette with monocyte/macrophage-specific elements to the CCR5 safe-harbor site. The engineered cells were able to produce glucocerebrosidase-expressing macrophages and preserve continuing repopulation and multi-lineage differentiation ability upon successive transplantation [28]. In a more recent study, CRISPR-based editing of the homozygous L444P (1448T→C) *GBA* mutation in human-induced pluripotent stem cells led to creation of both heterozygous and homozygous isogenic lines. Experiments in these lines confirmed that correction of the induced mutation reestablishes normal macrophage functions, as demonstrated by enzyme activity assay as well as motility, and phagocytosis assays. On the other hand, infection of GBA−/−, GBA ± and GBA+/+ macrophages with the Mycobacterium tuberculosis demonstrated correlation between impaired mobility/phagocytic activity and decreased level of bacterial engulfment and replication. Thus, GD might have a protective effect against tuberculosis [29].

**Table 2:** Application of CRISPR for correction of the *GBA* mutation.

Corrected/Engineered cells	Gene	locus	Delivery system	Analysis of targeting efficiencies	Features of HSCTs	Features of HPSC derived macrophage/monocyte	Methodology Summary	References
HPSC	glucocerebrosidase expression cassettes (CCR5 safe harbor locus)	–	AAV 1. Spleen Focus-Forming Virus 2. CD68S promoter (CD68S-GCase-P2A-Citrine)	Percentage of cells that are positive for Citrine (Citrine+) and the percentage of CCR5 alleles that have on-target cassette integrations through molecular analysis.	HPSCs cultured using this method displayed typical ameboid shape and expressed monocyte/macrophage lineage markers CD14 and CD11b while showing reduced levels of the HPSC marker CD34.	Human HPSCs edited with SFFV-GCase-P2A-Citrine and CD68S-GCase-P2A-Citrine constructs generated macrophages displaying Citrine expression, typical morphology, and effective phagocytosis (normal phagocytosis of pHrodolabeled E. coli). Analysis of CD14 and CD11b markers in mock-treated and Citrine ± populations indicated similar expression levels to unmodified cells, except for elevated expression in CD68S-GCase-Citrine + cells under both HPSC and macrophage differentiation conditions	1. rAAV vector plasmid construction 2. rAAV production 3. HPSC isolation and culturing 4. Gene editing in HPSCs 5. Assessment of cassette integration using ddPCR 6. Colony-forming unit assay and clonal genotyping 7. Macrophage differentiation and flow cytometry 8.Phagocytosis assay 9. Transplantation of CD34^+^ HPSCs into NSG Mice 10. Assessment of human cell engraftment 11. Glucocerebrosidase activity assay 12. Immunocytochemistry and imaging 13. Tissue macrophage isolation	[28]
hi-PSC	glucocerebrosidase	1448T→C mutation in exon 10 of GBA	AAV	Sequencing of 8 sites closely related to the sgRNA target	Pluripotency marker expression was validated through immunostaining with antibodies targeting Oct4, Sox2, Tra-1-60, Tra-1-81, and DAPI. Karyotypes of the GBA ± and GBA+/+ hiPSCs was normal.	Elevated pro-inflammatory markers in GBA mutant macrophages suggest a link to disease susceptibility, while phagocytic function shows improvement post-correction of the mutation, indicating restored capacity. HiPSC-derived macrophages with corrected GBA mutations showed heightened vulnerability to M. tuberculosis infection.	1. CRISPR Correction of GBA Mutation in Pluripotent Stem Cells 2. Macrophage Functional Assays 3. Macrophage Infection Assay Using Mycobacterium tuberculosis 4. Screened for GBA mutation using *Nci*I digestion and confirmed genotypes through sequencing of amplified DNA in hiPSCs	[29]

## 4 Discussion

The advent of genome editing technologies, particularly the CRISPR/Cas9 method, has offered the opportunity for the establishment of cellular and animal models of inherited diseases through introduction of site-specific mutations within the corresponding genes. This technology has been exploited in the field of GD for the purpose of high-throughput screening of therapeutic substances, identification of the underlying mechanism of their action on different phenotypic features, and providing insight into the pathologic cascades that are involved in GD [19]. Different cell lines, including human embryonic kidney (HEK) 293, human glioblastoma cell line U87, human monocytic cell line THP-1, haploid human cell line HAP1, human fibroblast cell line hFB and neuroblast cell line BE(2)-M17 have been the subjects of this type of intervention. HAP1 has the advantage of being near haploid, thus genes are hemizygous and so, phenotypes are instantly visible following induced mutations enabling examination of the effects of specific mutations in a human-related context [30]. Meanwhile, manipulation of THP-1 and U87 cells has facilitated development of GD monocyte and glia, respectively [18], thus identification of the effects of *GBA1* mutations in the corresponding tissues.

The molecular basis of pathogenesis of certain mutations has also been investigated using this technique [20]. This technique can also been used for testing personalized therapeutic options according to genetic basis of each patient. Moreover, CRISPR/Cas9 has facilitated recognition of involvement of other genetic abnormalities in the pathogenesis of metabolic disorders such as GD and investigation of the complex molecular mechanisms underlying this disorder and the regulation of β-glucosidase activity [22].

## 5 Conclusion

Although this technique has an inherent therapeutic property, this issue has been less studied in the context of GD. However, CRISPR/Cas9-engineered human hematopoietic stem cells exhibited monocyte/macrophage-specific expression of glucocerebrosidase [28] and proved to be applicable in the treatment of GD. Thus, this field needs to be explored in future studies.

Finally, based on the involvement of *GBA* mutations in the pathogenesis of Parkinson's disease, CRISPR/Cas9-based modeling systems have been used to show the importance of lysosomal function and lipid metabolism in the pathogenesis of this neurodegenerative disorder [23]. Thus, these modeling systems have a wide array of application in mechanistical studies ranging from a metabolic condition to a neurodegenerative disorder of aging.

## CRediT authorship contribution statement

**Mehran Reyhani-Ardabili:** Data curation, Conceptualization. **Mohadeseh Fathi:** Visualization. **Soudeh Ghafouri-Fard:** Writing – review & editing, Writing – original draft, Supervision, Conceptualization.

## Ethics approval and consent to participate

Not applicable.

## Consent for publication

Not applicable.

## Availability of data and material

Not applicable.

## Funding

No funding was received.

## Declaration of competing interest

The authors declare that they have no known competing financial interests or personal relationships that could have appeared to influence the work reported in this paper.
